# Efficacy and Safety of Orogastric vs. Nasogastric Tube Feeding in Preterm Infants: A Systematic Review and Meta-Analysis

**DOI:** 10.3390/children11111289

**Published:** 2024-10-25

**Authors:** Huazi Liu, Qiang Fei, Tianming Yuan

**Affiliations:** Department of Neonatology, Children’s Hospital, School of Medicine, Zhejiang University, National Clinical Research Center for Child Health and Disease, Hangzhou 310052, China; 21718452@zju.edu.cn (H.L.);

**Keywords:** oral/nasal route, preterm infants, growth, adverse events, meta-analysis

## Abstract

Background: Enteral nutrition can be delivered to the stomach using nasogastric or orogastric tubes, with each route having advantages and disadvantages. This meta-analysis aimed to compare the effects of these methods on growth, development, and the incidence of adverse outcomes. Methods: This analysis included studies that enrolled preterm infants who received nasogastric or orogastric tube feeding. We searched databases including PubMed, Embase, Web of Science, and the Cochrane Central Register of Controlled Trials. Only randomized controlled trials were selected. We used version 2 of the Cochrane tool to assess the risk of bias in randomized trials and Review Manager 5.4 software to perform the meta-analysis. Results: Six studies involving 265 preterm infants were included. The meta-analysis showed that orogastric tube feeding took significantly longer to establish full enteral tube feeding compared to nasogastric tube feeding (MD = 1.62, 95% confidence interval [CI]: 0.99–2.26, Z = 5.02, *p* < 0.01). However, no significant difference was observed between the two groups regarding time to regain birth weight (MD = −0.38, 95% CI: −2.2–1.44, Z = 5.02, *p* = 0.68). Data on adverse events were insufficient to perform a combined analysis. Conclusions: Preterm infants fed via nasogastric tubes took less time to reach full enteral feeding than those fed via orogastric tubes. Further research is required to evaluate the effect of feeding routes on adverse outcomes.

## 1. Introduction

In preterm or low-birth-weight infants, enteral feeding is challenging due to poorly coordinated sucking and swallowing, neurological immaturity, and respiratory distress [1]. Early enteral feeding can enhance micronutrient delivery, stimulate intestinal microbiome development and intestinal maturation, and promote neurodevelopment [2]. A correlation may exist between the time to regain birth weight and the severity of prematurity-associated retinopathy [3] and neonatal growth velocity [4]. In preterm infants, extrauterine growth restriction poses a risk of poor neurodevelopmental outcomes [5,6].

Enteral feeding can be delivered using nasogastric (NG) or oral–gastric (OG) tubes. NG tubes are easier to secure; however, they can increase nasal airway resistance by 50%, potentially leading to long-term issues such as respiratory muscle fatigue and weight gain [7,8]. Moreover, pharyngeal airway collapse can be caused by increased airway resistance, increasing prematurity-related apneas [9]. In contrast, OG tube feeding may reduce the risk of apnea in premature infants since they are obligate nasal breathers. However, securing OG tubes is more challenging, with a higher risk of dislodgment and the occurrence of palatal grooves [10,11]. To date, two systematic reviews have examined the effectiveness of NG and OG tube feeding in preterm infants [1,11]. These reviews included only two or three trials, providing insufficient data to guide clinical practice. This meta-analysis included more trials to evaluate the impact of NG and OG tube feeding on growth and development and to assess the incidence of adverse events in preterm infants.

## 2. Materials and Methods

### 2.1. Search Strategy

The current protocol has been registered in the PROSPERO database (registration number (CRD42024536313) and is reported according to the reporting guidance provided in the Preferred Reporting Items for Systematic Reviews and Meta-Analyses (PRISMA) statement [12].

A comprehensive literature search was conducted on the electronic databases PubMed, Embase, Web of Science, and the Cochrane Central Register of Controlled Trials (CENTRAL) with no language restrictions from April 2024 onwards. Synonyms were identified using the Medical Subject Headings (MeSH) database. The full search strategy used for PubMed was as follows: (((((((Intubation, Gastrointestinal [MeSH Terms]) OR (Gastrointestinal Intubation*[Title/Abstract])) OR (Intubation*, Gastrointestinal [Title/Abstract])) OR (Intubation*, Nasogastric [Title/Abstract])) OR (Nasogastric Intubation*[Title/Abstract])) OR (nasoenteral [Title/Abstract])) OR (nasal [Title/Abstract])) AND (((orogastric [Title/Abstract]) OR (oral [Title/Abstract])) OR (oroenteric [Title/Abstract])) AND (((((((Infant, Premature[MeSH Terms]) OR (Infants, Premature[Title/Abstract])) OR (Premature Infant*[Title/Abstract])) OR (Infant*, Preterm[Title/Abstract])) OR (Preterm Infant*[Title/Abstract])) OR (Neonatal Prematurity[Title/Abstract])) OR (Prematurity, Neonatal[Title/Abstract])). A modified version of this search strategy has been developed for use with other electronic databases.

### 2.2. Inclusion and Exclusion Criteria

The inclusion criteria were based on the PICOS framework.

(I) Participants: preterm infants (<37 weeks gestation) who can breathe spontaneously and need gastric tube feeding.
(II) Intervention: the intervention group received OG tube feeding.
(III) Control: the control group received NG tube feeding.
(IV) Outcomes:
  Primary outcomes:
  - Establishment of full enteral tube feeds (days): at least 140–160 mL/kg/day;
  - Time to regain birth weight (days).
  Secondary outcomes:
  - Apnea: breathing pauses that last for more than 10 s;
  - Bradycardia: heart rate < 100/min.
  Desaturation: SpO_2_ < 85%.
  - Tube displacement: displacement or removal of a feeding tube without intention.
  - Aspiration pneumonia: radiological and/or clinical evidence of lower respiratory tract compromise caused by aspiration of stomach contents covertly or evidently [13];
  - Gastric residuals: gastric residual volume > 50% of the previous meal [14];
  - Necrotizing enterocolitis: any stage (modified Bells staging) [15].
(V) The type of study: randomized controlled trials (RCTs).

Infants with congenital gastrointestinal or chromosomal anomalies, necrotizing enterocolitis, neurological abnormalities, or requiring mechanical respiratory support were not included in this meta-analysis. We also excluded systematic reviews, reviews, case reports, news, editorials, and patents.

### 2.3. Study Selection and Date Extraction

Following the screening of the article titles and abstracts, two reviewers (H. Liu and S. Zhou) obtained the full-text of relevant studies and extracted data based on the inclusion and exclusion criteria. Two evaluators extracted the following information: authors, year, group, number of participants, gestational age, postnatal age, birth weight, intervention, following up, and outcome indicators. The methods of randomization, allocation, and blinding used in the included studies were also extracted.

### 2.4. Assessment of Risk of Bias

Using Version 2 of the Cochrane tool for assessing bias in randomized trials (ROB 2), the risk of bias was assessed for the included studies. Five domains, i.e., randomization process, effect of assignment to intervention, missing outcome data, measurement of the outcome, and the selection of the reported result, were assessed. The bias risk of each domain could be categorized into three levels: low risk, some concern, and high risk [16]. All disagreements were resolved through discussion with a third party until consensus was achieved.

### 2.5. Statistical Analysis

We used Review Manager 5.4 software to perform the meta-analysis. The mean difference (MD) was used to estimate the effect for continuous variables and 95% confidence intervals (CI) were calculated. Heterogeneity among studies was estimated with the I^2^ statistic, with I^2^ of 25–50%, 50–75%, and >75% representing low, moderate, and high heterogeneity, respectively. If the heterogeneity was low (I^2^ < 50%), a fixed-effects model was used. If the heterogeneity was high (I^2^ ≥ 50%), a random-effects model was used. Sensitivity analyses were performed to explore the sources of heterogeneity.

## 3. Results

### 3.1. Literature Search and Screening

**Figure 1:**
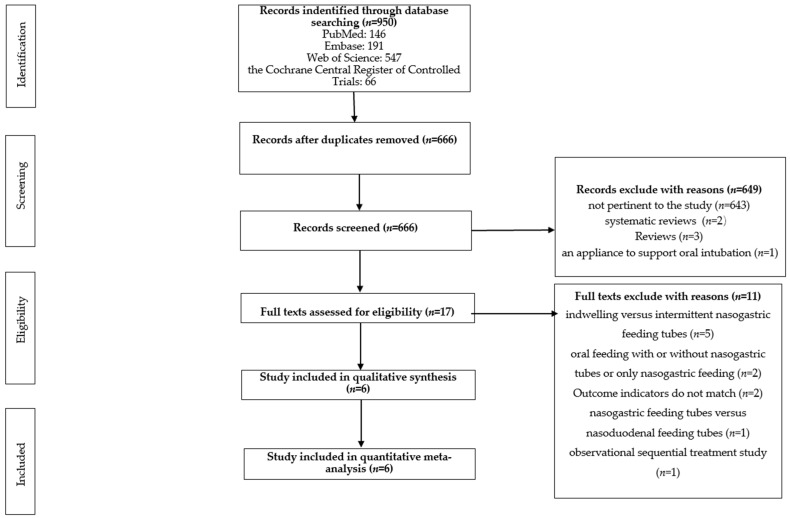
Literature screening flow chart.

A total of 950 studies were identified from the database, of which 284 were duplicate records. Among the remaining 666 study records, there were 643 irrelevant studies, two systematic reviews, three reviews, and one study on an appliance to support oral intubation. We read the full text of 17 articles, and excluded 11 for the following reasons: indwelling vs. intermittent nasogastric feeding tubes (*n* = 5), oral feeding with and without NG tubes or only NG tubes (*n* = 2), NG feeding tubes vs. nasoduodenal feeding tubes (*n* = 1), observational sequential treatment study (*n* = 1), and some studies that did not match the outcome indicators outlined in PICOS (*n* = 2). This meta-analysis ultimately included six studies [17,18,19,20,21,22]. Figure 1 shows the study screening process.

### 3.2. Basic Characteristics of Included Articles

The meta-analysis included six studies [17,18,19,20,21,22], with a total of 265 infants (158 with an OG tube and 160 with an NG tube). The study by Bohnhorst et al. [19] was a randomized controlled cross-over trial in which feeding tubes were placed orally or nasally in 32 infants for 12 h each. The study by Badran et al. [21] enrolled 21 infants and labeled each feeding tube insertion episode as FTIE. These lasted from the time of tube insertion to the time of tube replacement. There were 80 FTIEs in the orogastric and nasogastric tube groups, respectively. Four studies [18,19,20,22] enrolled preterm infants whose gestational age (GA) was 32 weeks, and the other two studies enrolled preterm infants whose GA was >32 weeks [17,21]. The postnatal age of the infants when enrolled in the three studies ranged from day 1 to day 12.5 after birth [17,18,20]. The birth weight (BW) of the infants varied; most of them were below 1800 g at birth. The experimental group were fed via orogastric tubes, while the control group were fed via nasogastric tubes. The follow-up time of the studies varied because of different outcome indicators. The outcome indicators were the time to complete enteral feeding; regaining birth weight; the incidence of apnea, bradycardia, and desaturation; tube displacement; aspiration; gastric residuals; and necrotizing enterocolitis. The results are shown in Table 1.

### 3.3. Risk Assessment of Bias of Included Articles

**Figure 2:**
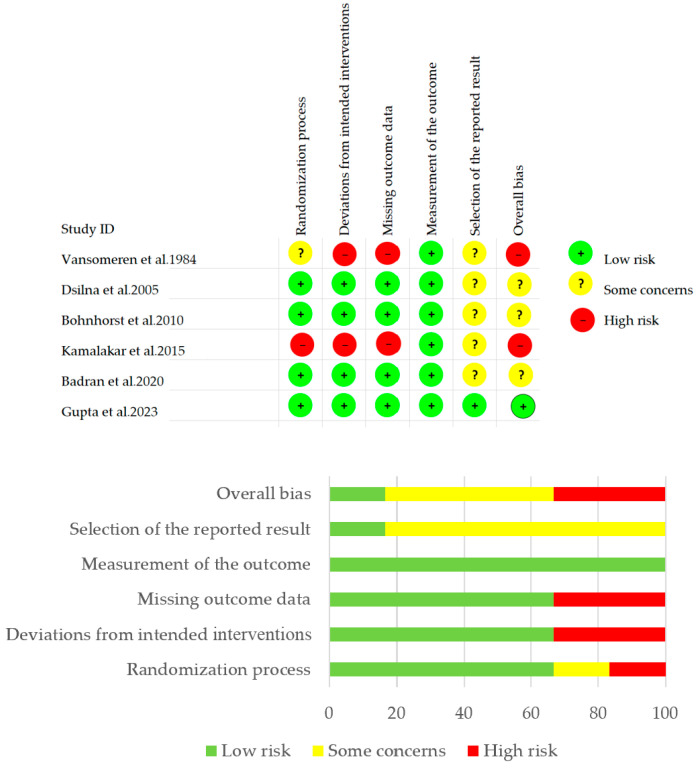
Risk of bias graph and summary [17,18,19,20,21,22].

We assessed the risk of bias of the included studies in five domains using ROB 2 [16]. Among the included studies, one had a low risk of bias, two had a high risk of bias, and three had some concerns about bias. When assessing risk of bias during the randomization process, there were four studies with low risk in which the allocation sequence was random and concealed until the participants were enrolled and assigned to interventions, one study with high risk because of a lack of detailed information for the randomization process, and one study with some concerns. Regarding deviations from the intended interventions, four studies had low risk, and two studies had high risk. In terms of a missing outcome date, four studies had a low risk of bias and two studies had a high risk. All studies had a low risk of bias regarding the measurement of the outcome. Five studies had some concerns regarding the risk bias, and one study had a low risk of bias regarding the selection of reported result. The results are shown in Figure 2.

### 3.4. Outcomes

#### 3.4.1. Time to Establish Full Enteral Tube Feeding (Days)

**Figure 3:**
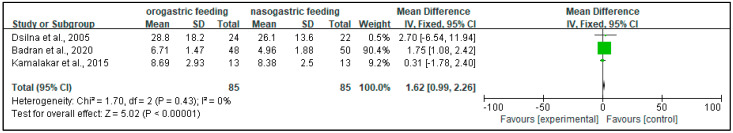
Forest plot of the time to establish full enteral tube feeding between two groups (days) [18,20,21].

There were three studies [18,20,21] that investigated the impact of the feeding tube on the length of time it took to establish full enteral tube feeding. They included 170 infants. Based on the heterogeneity test indicated by *p* = 0.43 and I^2^ = 0%, we used a fixed effects model. The meta-analysis showed that, compared to NG tube feeding, OG tube feeding took longer to establish full enteral tube feeding. The statistics showed a significant difference (MD = 1.62, 95% CI: 0.99–2.26, Z = 5.02, *p* < 0.01). The results are shown in Figure 3.

However, the definition for time to establish full enteral tube feeding was inconsistent in these three studies. Dsilna [18] and Badran [21] defined full enteral feeding as 140–160 mL/kg/day, while Kamalakar [20] defined full enteral feeding as 150 mL/kg/day.

#### 3.4.2. Time to Regain Birth Weight (Days)

**Figure 4:**
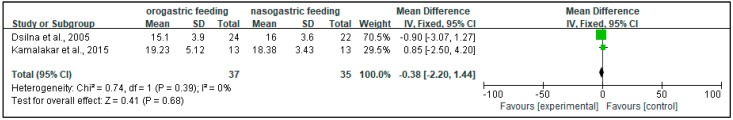
Forest plot of the time to regain birth weight between two groups (days) [18,20].

Three studies [18,20,21] reported the influence of the feeding tube on the time to regain birth weight. They included 170 infants. In the heterogeneity test, the *p*-value was 0.006 and the I^2^ value was 81%. The studies showed significant heterogeneity, so each study was excluded one by one, and the study [21] with the greatest impact on the heterogeneity was excluded at the end. The heterogeneity I*^2^* was 0% after exclusion, so the fixed effects model was used for analysis. The results showed that there was no significant difference in the time to regain birth weight between the OG tube feeding and NG tube feeding [MD = −0.38, 95% CI: −2.20–1.44, Z = 0.41, *p* = 0.68]. The results are shown in Figure 4.

#### 3.4.3. Apnea

Four trials [17,19,20,21] examined the effects of the feeding tube on apnea incidence and durations. Only the study by Bohnhorst et al. [19] used the same definition of apnea as us. They reported the episodes of apnea per hour, given as the median, with 95% confidence intervals. They did not find any significant differences ((0.7, 95% CI: 0.7–1.2) vs (0.84, 95% CI: 0.52–1.2, *p* = 0.33)). Vansomeren et al. [17] defined apnea as the absence of nasal airflow for five seconds or more. Badran et al. [21] defined it as breathing pauses lasting over 20 s, or at least 10 s in cases of bradycardia or oxygen deprivation. Kamalakar et al. [20] defined it as cessation of breathing for a period of 20 s or more.

#### 3.4.4. Bradycardias and Desaturation

Two studies [19,22] reported the influence of feeding tubes on the episodes of bradycardias and desaturation per hour. Gupta et al. [22] reported the mean and standard deviation of bradycardias and desaturation per hour. They found that the OG tube group had significantly fewer episodes of bradycardia and desaturations/hour than the NG tube group (0.24, 95% CI: 0.093–0.388, *p* = 0.002). Bohnhorst et al. [19] defined bradycardias as a fall in instantaneous heart rate by more than a third of an infant’s baseline heart rate and desaturation as SpO_2_ < 80%.

#### 3.4.5. Tube Displacement

Two studies [20,21] reported the influence of feeding tubes on the incidence of tube displacement in 124 preterm infants. Kamalakar et al. [20] expressed the values as mean and standard deviation. In the orogastric tube feeding groups, tube displacement (times/day) was higher than in the nasogastric tube feeding groups (−0.4462, 95% CI: −0.6996–−0.1927; *p* = 0.001). In a study by Badran et al. [21], the displacement rates of orogastric tubes were significantly higher than those of nasogastric tubes (*p* = 0.02).

#### 3.4.6. Aspiration Pneumonia

Kamalakar et al. [20] studied the influence of feeding tubes on aspiration in 26 preterm infants, but did not report the values or results. Badran et al. [21] reported these values as a percentage of the total; they found that the incidence rates of aspiration in OG was significantly higher in the OG tube group compared with the NG tube group (*p* = 0.02).

#### 3.4.7. Gastric Residuals

Dsilna et al. [18] studied the rate of gastric residuals among infants in 46 preterm infants, but they did not report values. There was no significant difference between the OG tube group and the NG tube group in terms of gastric residuals reported by Badran et al. [21].

#### 3.4.8. Necrotizing Enterocolitis

Kamalakar et al. [20] studied the incidence of necrotizing enterocolitis in preterm infants, but they did not report any values. According to Badran et al. [21], necrotizing enterocolitis did not differ significantly between infants with OG or NG tubes.

## 4. Discussion

As shown in this meta-analysis, NG tube feeding may reduce the amount of time needed to establish complete enteral tube feeding in preterm infants compared to OG tube feeding. Using NG tubes or OG tubes does not affect birth weight recovery time. Due to insufficient clinical data, we cannot determine the relationship between tube feeding route and adverse reactions. To date, two systematic reviews have examined the effectiveness of NG and OG tube feeding in preterm infants [1,11]. Moreover, these reviews included only two or three trials, providing insufficient data to guide clinical practice. We included three additional trials in our meta-analysis and conducted a quantitative analysis. This is the advantage of our study.

Enteral feeding and parenteral feeding are two methods of nutrient provision due to feeding intolerance in preterm infants. The use of parenteral nutrition is associated with an increased risk of parenteral-nutrition-associated cholestasis, retinopathy of prematurity, and catheter-associated bloodstream infection [23,24,25]. The early delivery of enteral feeding could provide essential micronutrients more quickly via fortification when compared with parenteral feeding alone [2]. In animal and human studies, enteral feeding can not only prevent intestinal villous atrophy, but can also promote intestinal development [26,27]. An observational cohort study showed that the intake of enteral protein, fat, and caloric intake were associated with larger volumes of cerebellum and basal ganglia. There is also a negative relationship between parental nutrition and the volume of cerebellum, gray matter, and basal ganglia, and total brain cerebellum and basal ganglia; this may indicate that enteral nutrition could enhance brain growth and neurodevelopment [28]. Our meta-analysis shows that NG tube feeding can shorten the time to full enteral feeding compared to OG tube feeding. The systematic review by Watson and Hawes [1,11] did not address whether NG or OG tubes affect time to enteral tube feeding. This is because only one study reported any results. A correlation may exist between the time to regain birth weight and the severity of prematurity-associated retinopathy [3], called neonatal growth velocity [4]. In preterm infants, extrauterine growth restriction poses a risk of poor neurodevelopmental outcomes [5,6]. OG tube feeding and NG tube feeding did not significantly differ in the time to regain birth weight based on our meta-analysis [MD = −0.38, 95% CI: −2.20–1.44, Z = 0.41, *p* = 0.68].

Compared to neonates suffering from necrotizing enterocolitis, those without the condition achieved full enteral feeding earlier [29,30]. Enteral feeding may be delayed by slow gastrointestinal development, interruptions in feeding, and antibiotic use [31]. Only two trials were included in our meta-analysis that examined the incidence of necrotizing enterocolitis in two different groups. Kamalakar et al. [20] did not report any data or draw a conclusion. Badran et al. [21] found no significant difference between the OG tube group and the NG tube group. There is a need for further research on whether there is a difference in the incidence of necrotizing enterocolitis between the two groups, and whether that difference is related to the time to full enteral feeding.

It is common practice to assess feed tolerance and guide feeding in preterm infants using gastric residual volumes. An increase in or alteration of gastric residuals can often serve as a warning sign of necrotizing enterocolitis. Our meta-analysis included only two studies that compared the gastric residual frequency between two groups [18,21]. No significant difference was found in the two trials. There have been more studies conducted in recent years investigating the rationale for routine gastric residual monitoring, which may result in interruptions of enteral feeding. A study by Parker et al. [32] involved 143 infants divided into two groups: the residual group and the group with no residuals. The researchers found that the no residual group received more enteral nutrition at weeks 5 (137.2, 95% CI: 128.6–145.8, *p* = 0.03) and 6 (141.6, 95% CI: 133.2–150.0, *p* = 0.03) after birth. In both groups, the odds of necrotizing enterocolitis were similar. In Tomas et al.’s [33] study, 87 infants were divided into two groups, one that underwent routine gastric residual volume assessment and one that was subject to gastric residual volume measurement when they had feeding intolerance. The researchers found no differences in the time to achieve full enteral feeding, incidence of necrotizing enterocolitis, sepsis, and regain birth weight between the study and control groups. Recent systematic reviews [34] compared the routine monitoring of preterm infants’ gastric residuals with no monitoring. According to the authors, monitoring gastric residuals increases the number of days of parenteral nutrition, the time it takes to establish full enteral feeding, and the risk of invasive infections. However, monitoring infants does not seem to have any effect on necrotizing enterocolitis. In the future, we can opt not to routinely monitor gastric residuals in trials involving preterm infants with enteral feeding tubes. There was no significant difference in the time to regain birth weight between NG tube and OG tube groups in this meta-analysis, and we could not draw any conclusions about the incidence of adverse events, including apnea, bradycardias, desaturation, tube displacement, aspiration, gastric residuals, and necrotizing enterocolitis because of insufficient clinical data.

There were several limitations to our study. First, the number of studies included in the meta-analysis was small, and the risk of bias of some of the included studies is high. Second, the definition of outcome index is inconsistent. Different studies define full enteral tube feeding differently. For example, Dsilna [18] and Badran [21] defined it as 140–160 mL/kg/day, while Kamalakar [20] defined it as 150 mL/kg/day. Third, there are only two outcomes that could be meta-analyzed. Data on adverse events were insufficient to perform a combined analysis.

## 5. Conclusions

In conclusion, our meta-analysis shows that NG tube feeding can shorten the time to full enteral feeding compared with orogastric tube feeding. To compare the growth and incidence of adverse events in preterm infants with naso-enteric feeding tubes and oro-enteric feeding tubes, larger randomized controlled trials are needed.

## Figures and Tables

**Table 1:** Basic characteristics of included studies.

Study	Group	Patients (*n*)	Gestational Age (Week)	Postnatal Age (d)	Birth Weight (g)	Intervention	Follow-Up	Outcome Indicators
Vansomeren et al., 1984 [17]	experiment group	20	31 (30–34) *	6 (2.5–12.5) *	1420 (1280–1740) *	OG feeding	14 days	(1) apneas (the number of episodes per hour on 3 days, 7 days)
	control group	22	32 (31–34) *	2 (1–6) *	1370 (1320–1760) *	NG feeding		
Dsilna et al., 2005 [18]	experiment group	24	26.8 (1.4) ^‡^	within 30 h of birth	899 (179) ^‡^	OG feeding	birth to postmenstrual age (32 weeks)	(1) time to achieve full enteral feeding (140 to 160 mL/kg/day) (2) time to regain birth weight (3) gastric residuals (the total number of the occasions)
	control group	22	26.6 (1.2) ^‡^		833 (177) ^‡^	NG feeding		
Bohnhorst et al., 2010 [19]	experiment group	32	29 (24–31) *	postmenstrual age < 36 weeks	1195 (465–1885) *	OG feeding	12 h	(1) apneas (the frequency of episodes per hour) (2) bradycardia and desaturation (the frequency of episodes per hour)
	control group					NG feeding		
Kamalakar et al., 2015 [20]	experiment group	13	<32	between day 1 and day 7 of birth	<1500 g	OG feeding	until infants achieved full enteral feeds	(1) time to achieve full enteral feeding (at least 150 mL/kg/day) (2) time to regain birth weight (3) incidence of apnea, tube displacement, aspiration, necrotizing enterocolitis
	control group	13				NG feeding		
Badran et al., 2020 [21]	experiment group	48	33.27 (1.08) ^‡^	postmenstrual age range 30–35 weeks	1753.3 (414.51) ^‡^	OG feeding	until infants achieved full enteral feeds	(1) time to achieve full enteral feeding (140 to 160 mL/kg/day) (2) time to regain birth weight (3) incidence of apnea, tube displacement, aspiration, necrotizing enterocolitis, bradycardia, desaturation, gastric residuals
	control group	50	33.32 (1.57) ^‡^		1859.6 (307.05) ^‡^	NG feeding		
Gupta et al., 2023 [22]	experiment group	21	≤32	postmenstrual age was 31.3 (1.8) ^‡^	1112.5 (450) ^‡^	orogastric feeding	from the time of insertion till the time needed to be changed	(1) bradycardia and desaturations (episodes per hour)
	control group					nasogastric feeding		

* Values are expressed as medians, and interquartile range is shown in parentheses. ^‡^ Values are mean (standard deviation).
